# Better Not to Know? Emotion Regulation Fails to Benefit from Affective Cueing

**DOI:** 10.3389/fnhum.2016.00599

**Published:** 2016-11-25

**Authors:** Siwei Liu, Marie-Anne Vanderhasselt, Juan Zhou, Annett Schirmer

**Affiliations:** ^1^Centre for Cognitive Neuroscience, Duke-NUS Medical SchoolSingapore, Singapore; ^2^Psychopathology and Affective Neuroscience Lab, Ghent UniversityGhent, Belgium; ^3^Department of Psychology, National University of SingaporeSingapore, Singapore; ^4^LSI Neurobiology/Ageing Programme, National University of SingaporeSingapore, Singapore

**Keywords:** reappraisal, emotion regulation, neuroimaging, principal component analysis

## Abstract

Often we know whether an upcoming event is going to be good or bad. But does that knowledge help us regulate ensuing emotions? To address this question, we exposed participants to alleged social feedback that was either positive or negative. On half the trials, a preceding cue indicated the feedback’s affective quality. On the remaining trials, the cue was uninformative. In two different blocks, participants either appraised feedback spontaneously or down-regulated ensuing emotions using a controlled appraisal strategy. Event-related potentials (ERPs) recorded throughout both blocks revealed an increased late positive potential (LPP) during cue and feedback epochs when cues were affectively informative as compared to uninformative. Additionally, during feedback epochs only, informative, but not uninformative, cueing was associated with an appraisal effect whereby controlled appraisal reduced the LPP relative to spontaneous appraisal for negative feedback. There was an opposite trend for positive feedback. Together, these results suggest that informative cues allowed individuals to anticipate an emotional response and to adjust emotion regulation. Overall, however, informative cues seemed to have prolonged and intensified emotional responding when compared with uninformative cues. Thus, affective cueing appears to be contraindicated when individuals aim to reduce their emotions.

## Introduction

Although emotions are generally useful, there are many situations in which we must regulate them by trading off a present against a future feeling and preventing a present feeling from becoming too strong (Schirmer, 2014). For example, we should probably avoid savoring a chocolate cake if this pleasure is followed by despair over gaining weight. Research on emotion regulation has shown the usefulness of cognitive strategies like reappraisal (Gross, 1998). However, little is known about how these strategies are shaped by prior knowledge about the emotional significance of an upcoming event. Here, we sought to address this issue and to explore the temporal course of affective (i.e., good vs. bad) cueing effects on emotion regulation by means of event-related potentials (ERPs). In the following sections, we review extant research on cued emotional responding, identify open questions, and develop our study goal.

### Cues to Regulate and their Effect on Emotional Responding

Several studies on cued emotional responding focused on the role of regulation cues signaling an impending emotional event that requires emotion regulation. These studies typically presented participants with instructions to increase, decrease, or maintain an emotional response evoked to a subsequent stimulus. Functional magnetic resonance imaging (fMRI) experiments showed that cueing participants to regulate as compared to simply experience an emotion increases activity in prefrontal regions while reducing ensuing subjective emotions (Vanderhasselt et al., 2013; Denny et al., 2014).

In ERP studies, regulation cues have been linked to a centro-parietal positivity called the late positive potential (LPP). This component was explored, not for regulation cues directly, but for the stimuli following such cues. The instructions to increase or decrease an emotion were found to increase and decrease the LPP, respectively (Hajcak and Nieuwenhuis, 2006; Langeslag and van Strien, 2013; for a review see Hajcak et al., 2010). Because of this and the fact that emotional relative to neutral stimuli increase the LPP, its amplitude is considered to reflect the intensity of an emotional response and, correspondingly, emotion regulation success (Hajcak et al., 2010). Note, however, that the LPP responds to a range of stimulus and task conditions. In the context of emotion, it is modulated by both the valence (i.e., pleasantness and unpleasantness) of a stimulus and the arousal the stimulus elicits (Schupp et al., 2000) and seems linked to activity in a range of cortical and subcortical regions (Sabatinelli et al., 2013). In the context of non-emotional paradigms, the LPP is modulated by stimulus salience, expectancy and task relevance among others (Johnson and Donchin, 1978; Kok, 2001). Therefore, many speculate that it reflects a range of emotional and perhaps more general cognitive processes including emotion discrimination, motivational relevance and resource allocation (Kok, 2001; Schupp et al., 2006; Hajcak et al., 2010; Sabatinelli et al., 2013).

Together, both fMRI and ERP research on regulation cues demonstrates that, if given the opportunity, individuals can initiate top–down processes before an upcoming emotional event and down-regulate ensuing emotions.

### Affective Cues and their Effect on Emotional Responding

Another approach to study cued emotional responding is to present participants with predictive stimuli that forecast the valence of an upcoming event. In the context of fMRI, it was shown that this form of affective cueing elicits brain activity in emotion regions such as the amygdala or the ventral striatum and that instructions to subdue emotional responses subdue this activity (Kalisch et al., 2005; Herwig et al., 2007; Delgado et al., 2008; Denny et al., 2014; Yoshimura et al., 2014).

Correspondingly, ERP studies found that affectively informative cues increase the LPP if they predict an emotional as compared to a neutral stimulus (Kolassa et al., 2005; Kopp and Altmann, 2005; Miltner et al., 2005; Michalowski et al., 2009, 2014). For example, in a study by Michalowski et al. (2015), a colored fixation cross informed participants whether an upcoming picture would be neutral, unpleasant, or contained a spider. Spider phobics, as well as control participants, responded with greater LPPs to unpleasant and spider pictures as compared to neutral pictures and this effect was preceded by an analogous response to cues. Importantly, there is evidence that the LPP enhancement to both cues and targets can be dampened by emotion regulation (Herbert et al., 2013; Langeslag and van Strien, 2013; Galli et al., 2014).

Together, both fMRI and ERP research suggests that affective cues may elicit a pre-emptive emotional response, as well as associated regulatory processes.

### The Present Study

While past research sheds light on the role of preparatory processes in emotion regulation, our understanding remains incomplete. For one, it remains unclear under which conditions affective cues facilitate regulatory responses to an emotion target. Existing research failed to directly address this issue. Either target responses were not investigated, or they could not be dissociated from cue responses because cues were always affectively informative. In other words, cues reliably indexed an upcoming affective challenge and were not compared to an uninformative cue condition.

Second, the emotion stimuli of past studies had low self-relevance and were typically heterogeneous (e.g., pictures involving other humans, animals, man-made objects or natural scenes) such that cueing was necessarily generic (Vanderhasselt et al., 2013; Denny et al., 2014). To the best of our knowledge, cued emotion regulation has never been explored using social feedback as a stimulus, which addresses these issues and is something participants encounter regularly outside the laboratory.

With these points in mind, we explored whether and how affective cues help individuals regulate emotional responses to social feedback. We developed a paradigm involving *regulation* and *affective cues* that preceded social feedback regarding the participants’ characteristics/looks. Regulation and affective cue manipulations were combined in the following way. In two separate blocks, cues prompted participants to appraise upcoming feedback either spontaneously or in a controlled manner aimed at down-regulating emotions (e.g., this person does not know me). The latter strategy compares with what is termed cognitive reappraisal in the literature (Gross, 1998). However, in our case emotion regulation is planned and initiated pro- rather than retrospectively, so we refer to this as controlled appraisal instead. Within each block, cues were either informative and revealed the valence [i.e., praise (positive)/critique (negative)] of the following target or they were uninformative.

Our goal was to test the following hypotheses. First, we expected controlled appraisal to elicit more effortful stimulus processing during the cue phase and to reduce emotional responding during the target phase. Accordingly, cues were expected to elicit larger LPP amplitudes, whereas targets were expected to elicit smaller LPP amplitudes for controlled relative to spontaneous appraisal. Second, and more importantly, we hypothesized that informative relative to uninformative cues would be more effective in enabling appropriate emotion regulation. Moreover, given that individuals may be naturally inclined to down-regulate negative more than positive emotions (Delgado et al., 2008), we expected negative informative cues to be more potent than positive informative and uninformative cues in motivating controlled appraisal. As such, we anticipated controlled appraisal effects reflected by the LPP to be largest on trials with negative informative cues.

## Materials and Methods

### Participants

Twenty-three female participants were recruited via posters. We focused on female participants only because of previous reports concerning sex differences in emotion (Schirmer, 2013) and the assumption that women might be more sensitive than men toward feedback about their looks. Three of the participants were excluded from data analysis due to excessive artifacts in the EEG. The remaining 20 participants (mean age 22.2 years, *SD:* 1.64) reported being free of hearing or visual impairments, having no history of depression or neurological conditions, and using their right hand for writing. Participants signed informed consent prior to participation in this study, which was approved by the Institutional Review Board of the National University of Singapore. They received S$30 as a token of appreciation.

### Materials

The stimulus material consisted of *cues* and *targets*. A positive emoticon :-), a negative emoticon :-(, and a question-mark served as cues. Targets comprised social feedback stimuli especially developed for the current study so as to address potential cross-cultural issues arising from the use of existing stimuli developed for Western populations. The targets were created as follows. One-hundred and twenty positive and 120 negative words describing a personality or physical characteristic were subjected to a valence rating by 16 participants (eight females) not included in the experiment proper. Raters judged word valence, arousal, and everyday usage. Valence was rated on a five point scale ranging from -2 (very negative) to +2 (very positive). Arousal was rated on a scale from 1 (not arousing) to 5 (extremely arousing). Finally, everyday usage was explored with the question “Would you say that this word can be used to describe a person? No (1) or Yes (2)?” Based on these ratings, separate word groups containing 90 positive (valence rating: *M* = 1.16; *SD* = 0.26; arousal ratings: *M* = 2.76; *SD* = 0.26; usage: *M* = 1.93; *SD* = 0.09) and 90 negative (valence rating: *M* = -1.12; *SD* = 0.29; arousal ratings: *M* = 2.24; *SD* = 0.30; usage: *M* = 1.92; *SD* = 0.06) words were selected. Word groups were matched for valence strength (i.e., difference from 0), arousal, and everyday usage as well as word length and word frequency (Balota et al., 2007). During the experiment, five high frequency words were repeated six times both within the positive and the negative word group as one might expect that such frequent words would be repeated across the different evaluators. Thus, the total number of word presentations for each word group was 120.

Selected emotion words were supplemented by a prelude such as ‘you look’ or ‘you are’ (e.g., you are attractive). Preludes used in this research were equally distributed across positive and negative feedback and could hence not bias emotional responses. Preludes and emotion words were presented individually but in immediate succession. Relevant for the present study were responses to targets only.

Although, it would have been beneficial to include neutral targets along with the selected positive and negative ones, we refrained from doing so because the present paradigm was already fairly ambitious involving two sessions with the latter one lasting over an hour. We hence feared that fatigue would dilute responses later in the experiment. Furthermore, replacing the positive or the negative feedback with neutral feedback seemed unviable. We considered it ethically problematic to present negative feedback only and we assumed that most feedback people give and receive is indeed emotionally charged and of mixed valence.

### Procedure

This study comprised two phases separated by two days to one week. An initial phase was conducted via email and served to frame the study as exploring first impressions. Participants were informed that they would exchange a personal photo and personal assessments with participants in an overseas lab. After participants submitted their own photo, they received a PowerPoint presentation containing 20 photos of bogus overseas individuals. For each individual, they came up with one or two simple adjectives concerning their physical and personal appearance (e.g., smart) and emailed those back to the experimenter. Participants were told that the experimenter would send their impressions to the overseas participants and in return receive impressions from overseas participants for use in the second phase of the study.

The second phase required participants to came to the lab. They were prepared for the EEG recording and received task instructions. They were informed that there would be two blocks during which they would see cues followed by the social feedback from the overseas participants about their own photo. One was a *spontaneous appraisal block* during which they should view cues and feedback without changing or specifically focusing on their emotions. The other was a *controlled appraisal block*, during which cues prompted them to initiate a cognitive strategy to down-regulate emotions ensuing from subsequent targets.

Following these instructions, participants practized the controlled appraisal together with the experimenter who asked them to express their thoughts verbally and coached them in case of problems. If necessary, participants were provided with example strategies including thoughts such as “the critique/praise of this person can’t be applicable, (s)he doesn’t know me at all, (s)he might know an egoistic person who looks like me, and (s)he might automatically attribute these characteristics to me.” The practice ended once participants seemed comfortable with and able to use at least one cognitive strategy effectively. The participant was then asked to use this strategy on all trials in the controlled appraisal block.

**FIGURE 1:**
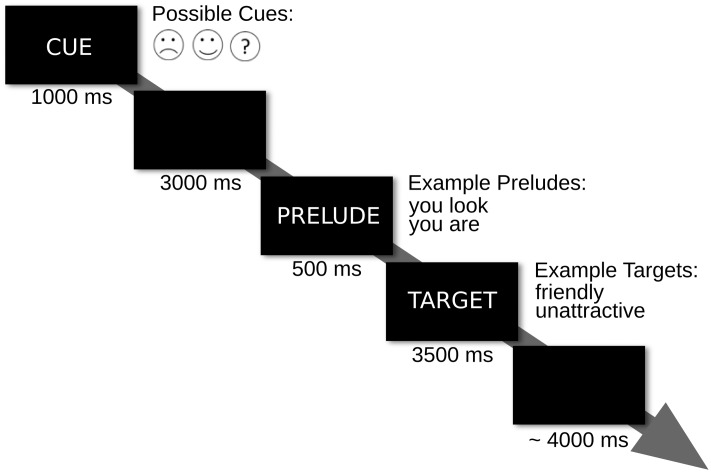
**Trial outline.** Trial events and their respective durations are illustrated.

The spontaneous appraisal and the controlled appraisal blocks each had 120 trials. Trials began with a 1 s cue. On 30 trials, the cue reliably predicted positive feedback and on another 30 trials, it reliably predicted negative feedback. On the remaining 60 trials, the cue was uninformative. Half of these trials entailed positive and the other half negative feedback. The cue was followed by a 3 s blank screen. Then a prelude was shown for 0.5 s followed by a target for 3.5 s. The inter-trial interval was jittered between 3.5 and 5.5 s in steps of 0.5 s (**Figure 1**).

The order of trials was pseudo-randomized such that trial valence was repeated no more than three times in a row. Participants took short breaks after completing 40 trials. The order of blocks was counterbalanced across participants.

After the experiment, participants were asked: (a) “What do you think is the purpose of this experiment?” and (b) “Do you think the evaluations were true?” Some participants expressed that not all the evaluations they saw truly reflected their personalities, as they were first impressions based on a photo. However, none of them reported not believing their authenticity.

### Electrophysiological Recording and Analysis

A 64-channel EEG cap with empty electrode holders was placed on the participant’s head. The electrode holders were filled with an electrolyte gel and the respective electrodes according to the modified 10–20 system. To measure the electrooculogram (EOG), individual electrodes were attached above and below the left eye and at the outer canthus of the right eye. Three electrodes were placed on left mastoid, right mastoid, and nose tip as alternative references. The data were recorded at 256 Hz with an ActiveTwo system from Biosemi, which uses a common mode sense active electrode for online referencing. An antialiasing filter was applied during data acquisition (i.e., sync filter with a half-power cutoff at 1/5 the sampling rate).

Electroencephalogram/electrooculogram data were processed with EEGLAB (Delorme and Makeig, 2004). The recordings were re-referenced to the nose and a 0.1–30 Hz bandpass filter was applied. The continuous data were visually scanned for non-typical artifacts caused by faulty channels, drifts or muscle movements and time points containing such artifacts were removed. Infomax, an independent-component analysis algorithm implemented in EEGLAB, was applied to the remaining data and components reflecting typical artifacts (i.e., eye movements) were removed. We then computed separate epochs for cues and targets using a 0.2 s prestimulus baseline and a 1 s ERP time window starting from stimulus onset. All epochs were baseline corrected and again visually screened for residual artifacts and re-referenced to the average of both mastoid electrodes (the prior nose-referencing enabled the detection of artifacts in all relevant channels including mastoids). ERPs were derived by averaging individual epochs for each participant and condition. An average of 26.2 (*SD*: 2.9) and 27.6 (*SD*: 2.6) epochs per condition entered the statistical analysis for cues and targets, respectively.

A traditional ERP analysis on average voltages of the LPP peak is presented in the Supplementary Materials. Here, we report the results of a temporal-spatial principal component analysis (PCA) that allows isolation of temporally and spatially overlapping ERP components (Kayser and Tenke, 2003; Dien, 2012). This approach has proved useful for a number of ERP components (Zhang et al., 2012; Luu et al., 2014; Zhou and Thomas, 2015) including the LPP elicited in emotion paradigms (Foti et al., 2009).

In a first analysis step, we performed a temporal PCA on the average ERP to reduce the data to relevant component time courses. Here, time points were the variables, while channels, conditions, and subjects were the observations (i.e., one observation refers to data in one channel, one condition, and one participant). The promax rotation method was used to separate variance orthogonally (Dien, 2010). Variance accounted for by each temporal component was calculated and sorted. Only temporal components that explained more than 1% of the total variance and with a time course similar to the LPP were selected. This reduced the number of analyses and helped control for multiple comparisons. Spatial PCA was then performed separately on the factor scores (FSs) obtained from each selected temporal component.

For the spatial PCA, channels served as variables while conditions and subjects served as observations. For each selected temporal component, we randomized the FSs to generate a random dataset. We then performed a 64-dimension spatial PCA on the randomized data set and calculated the variance explained by the resulting spatial components. We repeated this procedure 500 times and calculated the average explained variance. We also performed a 64-dimension spatial PCA on the original non-random FS data and calculated the explained variance of each resulting component. A scree plot was drawn to identify how many components had more explanatory power in the original compared to the average random data. The identified number of components became the number of dimensions in another spatial PCA on the original non-random FS data and Infomax was used as the rotation method (Dien, 2010). From the resulting spatial components for each time course (i.e., spatio-temporal components), we identified the one explaining the largest amount of variance and thus contributing most to the associated ERP. Finally, we selected the spatio-temporal components with a topography that overlapped with that of the typical centro-parietal LPP topography (see Supplementary Materials for a more detailed documentation).

The FSs of selected components for cue and target epochs were subjected to separate ANOVAs with *Appraisal* (spontaneous, controlled), *Affective Cueing* (yes, no), and *Target Valence* (positive, negative) as repeated measures factors. The Bonferroni method was applied in case multiple components were explored for a given ERP epoch. Specifically, as there were two components for cues, we adjusted our *p*-value to 0.025. As there was only one component for targets, we applied an uncorrected *p*-value of 0.05.

## Results

### Cue Epoch

**FIGURE 2:**
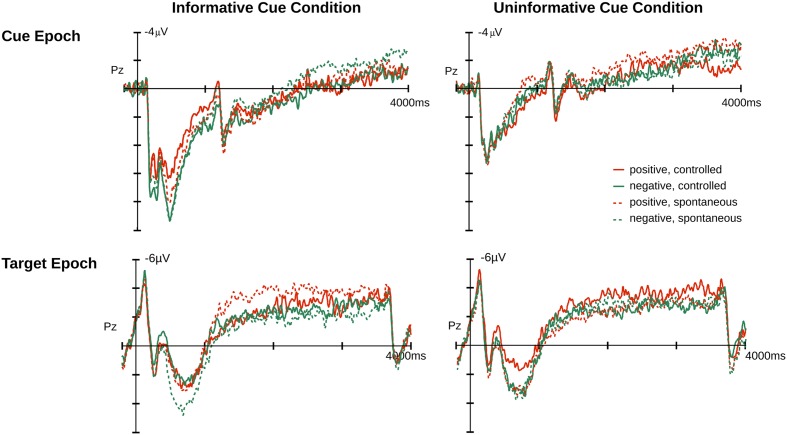
**Event-related potential (ERP) average at Pz for cue and target epochs.** Illustrated on the left is the ERP time course for the informative cue condition (affective cueing) and on the right for the uninformative cue condition.

**FIGURE 3:**
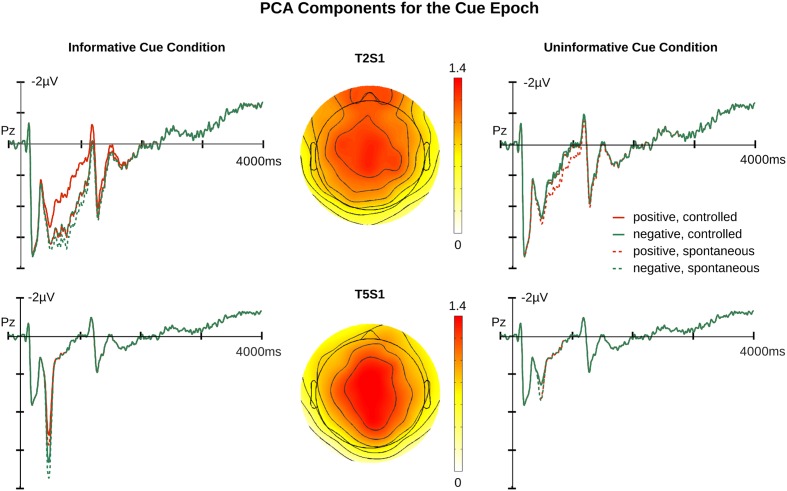
**Relevant principal component analysis (PCA) components for cue epochs.** Illustrated in the upper row are T2S1 traces and topography. Illustrated in the lower row are T5S1 traces and topography. The waveforms are the projection traces for each component at Pz. T2S1cue component peaked at 754 ms after cue onset. T5S1cue component peaked at 473 ms after cue onset. Additional time course information is available in the Supplementary Materials.

For the cue epoch, the temporal PCA produced seven principal components (PCs) that explained more than 1% of variance. Three temporal PCs peaked between 300 and 900 ms following cue onset and thus overlapped with the traditional LPP window (see Supplementary Materials). Accordingly, these components were subjected to spatial PCAs, two of which produced two temporal-spatial PCs with a central scalp distribution, referred to as T2S1cue and T5S1cue (T followed by a digit indicates the order of the temporal component, whereas S followed by a digit indicates the order of the associated spatial component). The FSs of both components were each subjected to an ANOVA using a Bonferroni corrected *p*-value of 0.025. **Figures 2** and **3** illustrate the original ERP and the back-projected ERP traces of T2S1cue and T5S1cue, respectively.

**FIGURE 4:**
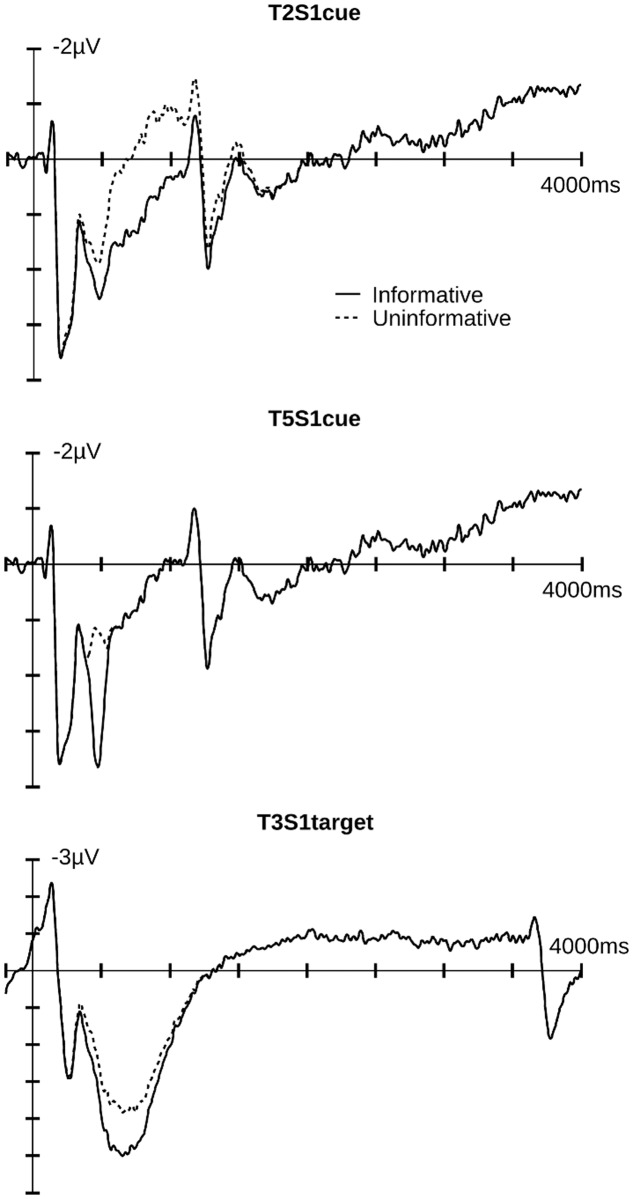
**Illustrated are the component traces for informative and uninformative cue conditions for both cue and target epochs.** T2S1cue and T5S1cue components peaked at 754 and 473 ms after cue onset, respectively. T3S1target component peaked at 833 ms after target onset. Additional time course information is available in the Supplementary Materials.

Analysis of T2S1cue yielded a significant main effect of *Affective Cueing* [*F*(1,19) = 7.84, *p* = 0.01] with no significant interactions (*p*s > 0.1). Informative cues elicited more positive responses in the LPP time window than did uninformative cues. T5S1cue also showed a significant main effect of *Affective Cueing* [*F*(1,19) = 47.21, *p* < 0.001]. Again, amplitudes were larger for the informative than the uninformative cue condition. These affective cueing effects are illustrated in **Figure 4**.

### Target Epoch

**FIGURE 5:**
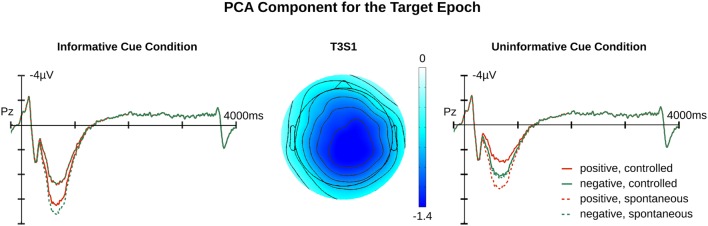
**Relevant PCA component for target epochs.** Illustrated are component traces and topography for T3S1target, the only LPP like component identified for targets. The waveforms are the projection traces at Pz for the informative and uninformative cue conditions. T3S1target peaked at 833 ms after target onset. Additional time course information is available in the Supplementary Materials.

For the target epoch, the temporal PCA produced five temporal PCs that explained more than 1% of variance. One PC showed a relevant temporal course overlapping with the LPP and was subjected to spatial PCA resulting in a temporal-spatial PC referred to as T3S1target that had a centro-parietal scalp distribution. The FSs of T3S1target were subjected to an ANOVA and follow-up comparisons made at *p* < 0.05. **Figures 2** and **5** illustrate the original ERP and the back-projected traces from T3S1target, respectively.

The main effect of *Affective Cueing* [*F*(1,19) = 17.52, *p* < 0.01] indicated that, contrary to our predictions, T3S1target amplitudes were larger in the informative relative to the uninformative cue condition. Additionally, there was an interaction of *Appraisal*, *Target Valance*, and *Affective Cueing* [*F*(1,19) = 6.24, *p* = 0.02]. Follow-up tests indicated that the *Target Valance* by *Appraisal* interaction was significant for the informative [*F*(1,19) = 7.21, *p* = 0.01], but not the uninformative cue condition [*F*(1,19) = 2.29, *p* = 0.15]. For the informative cue condition, the *Appraisal* effect was significant with negative feedback [*F*(1,19) = 5.1, *p* = 0.04] and marginal with positive feedback [*F*(1,19) = 3.869, *p* = 0.06]. Negative feedback following an informative cue elicited a smaller T3S1 amplitude in the controlled relative to the spontaneous appraisal condition. In contrast, positive target feedback following an informative cue tended to elicit a larger T3S1 amplitude in the controlled than the spontaneous appraisal condition.

## Discussion

The goal of this study was to determine whether and how affective cueing influences the processing of positive and negative social feedback under spontaneous and controlled appraisal conditions. We expected controlled appraisal to elicit more effortful stimulus processing during the cue phase and to reduce emotional responding during the target phase. Additionally, we hypothesized that informative cues, especially if forecasting negative feedback, would be more potent than uninformative cues in helping individuals regulate ensuing emotions.

The present results both confirm and contradict our hypotheses. First, looking at appraisal effects we found that, during the cue epoch, ERPs were comparable for the controlled and spontaneous appraisal conditions. Moreover, although appraisal effects during the target period reached significance, they were small and depended on both the information status of the cue and the valence of the target. Looking at affective cueing specifically, we found supporting evidence during the cue period. Informative cues elicited a greater LPP than uninformative cues, seemingly in-line with the idea of greater effortful processing. Surprisingly, however, this effect extended to the target period in that targets elicited a larger LPP following informative relative to uninformative cues. Moreover, a dissociation between positive and negative feedback conditions as a function of appraisal was found for targets only.

The remainder of this discussion will tackle the prominent differences between informative and uninformative cueing, offer an explanation for results that diverged from our hypotheses, and outline directions for future research.

### Informative vs. Uninformative Cueing

In the present study, cue type modulated the LPP during both cue and target epochs. During cue epochs, affectively informative cues elicited more positive potentials than uninformative cues. This effect repeated itself in the target epoch where it was further qualified by regulation instructions and feedback valence.

Focusing on the *cue epoch*, one may interpret our results as indicating that, compared to uninformative cues, informative cues recruited more processing resources allowing emotion regulation processes to be better prepared and targeted at specific feedback valence. However, the absence of an appraisal main effect and the fact that informative cues modulated cue and target responses similarly challenge this interpretation. Specifically, the absence of an appraisal effect contradicts the possibility that individuals effectively prepared appraisal processes to cues. Additionally, the failure of informative cue effects to be smaller for targets than cues raises the possibility that both reflect emotional processing instead. Most likely, informative cues already triggered an emotion allowing participants to anticipate what they would experience in response to the target. This possibility accords with existing evidence on cued affective responding (Kolassa et al., 2005; Kopp and Altmann, 2005; Miltner et al., 2005; Michalowski et al., 2009, 2014) and on the relation between LPP amplitude and emotion (Yang et al., 2012; Herbert et al., 2013; Langeslag and van Strien, 2013; Sarlo et al., 2013; Galli et al., 2014).

Focusing on the *target epoch*, our results speak to the influence of affective cueing on emotion regulation. The fact that targets following informative but not uninformative cues showed an appraisal effect, suggests that affective cueing promotes emotion regulation. Moreover, looking at the appraisal effect in the informative cue condition, we can furthermore conclude that negative and positive targets are influenced differently. Specifically, the anticipation of negative feedback allowed individuals to reduce their LPP in the controlled relative to the spontaneous appraisal condition. In contrast, the anticipation of positive feedback had a marginally opposite effect, possibly because individuals were less motivated to explicitly discount another’s positive view of themselves. In line with this, previous results show successful up- but not down-regulation of emotional responses to reward (Langeslag and van Strien, 2013).

Surprisingly, targets failed to elicit an appraisal effect in the uninformative cue condition. Superficially, this condition compares with the many emotion regulation studies that report an LPP decrease when participants down-regulate an emotional response. Hence, a similar effect, albeit smaller than in the informative cue condition, was expected here. That such an effect did not appear may relate to aspects of our paradigm that differ from prior work (for a review see, Hajcak et al., 2010). Specifically, we used self-relevant social stimuli for emotion elicitation that have not yet been used previously in the context of emotion regulation. Perhaps regulation efforts toward such stimuli, especially if they are positive, are generally weaker or more variable than those directed at more generic aversive images.

Together the effects observed to cues and targets in the present study suggest that knowing compared to being ignorant about the valence of an upcoming event “warms-up” individuals for an impending emotion. Moreover, affectively informative cues, but not uninformative cues, seem to elicit an emotional response that, if negative, individuals can wilfully subdue. Importantly, however, this response despite being wilfully subdued ends up being comparable, if not larger than, emotional responses following uninformative cues. Thus, affective cueing appears to be of little use when individuals aim to down-regulate their emotions.

### Directions for Future Research

Although, the present study introduced a novel methodological approach and reports interesting findings, it is not without limitations.

One such limitation is that interpretations hinge on an ERP component known to reflect a fairly general array of processes. Moreover, in the absence of additional measures of emotion regulation effort and/or success, the exact role of affective cueing remains unclear. Prior research has addressed this problem by recording subjective emotions felt after a block of trials (Hajcak and Nieuwenhuis, 2006) or after each trial (Vanderhasselt et al., 2013). Unfortunately, this approach was not feasible here. Due to the affective cue manipulation, positive and negative conditions could not be presented in blocks. Additionally, recording a behavioral response elicits a confounding positivity overlapping with the LPP. Thus, to implement an additional trial-based judgment, this judgment would have to be made after the target leading to a significant increase in study duration. Given our concerns about fatigue and that it may be doubtful as to whether trial-based judgments reliably and honestly reflect emotion, we decided against them and, like others, relied on ERPs only (Langeslag and van Strien, 2013).

A second methodological shortcoming concerns the cueing manipulation. In an attempt to minimize cognitive load, we used cues for which participants did not need to learn a mapping between the cue and the target valence. Thus, when presented with the cue, participants could immediately activate an emotion regulation strategy. The downside was that cues varied, albeit minimally, between conditions and that condition differences in the ERP may be confounded. However, given the absence of differences between positive and negative cues during the cue epoch and their differential modulation as a function of appraisal in the target epoch, small graphical differences between cue types may have been negligible.

Last, we would like to mention the role of individual differences for the present results. We conducted this study on a group of young women suspecting that they would be most sensitive toward feedback about looks (Schirmer, 2013). Yet, whether sex, age, and other factors like personality or culture are indeed relevant awaits future research. Moreover, it remains open whether and how the relationship between the sender and receiver of feedback matters. For example feedback from individuals of a different culture, as implemented here, may be less effective than feedback from one’s own culture.

## Conclusion

To summarize, we found support for the idea that prior knowledge about an upcoming event can promote emotion regulation success. Informative, but not uninformative, cueing reduced the LPP to negative social feedback during controlled relative to spontaneous appraisal. Given prior evidence for a reduction in LPP amplitude indicating emotion regulation success, we speculate that our results indicate that affective cueing reduces emotions to a regulatory target. Notably, however, both informative cues and affectively anticipated targets elicited a larger LPP than uninformative cues and unanticipated targets, suggesting that prior affective knowledge triggers a pre-emptive emotional response and increases feedback impact. Affective cueing, thus, produces mental and emotional costs that one may happily indulge in when things look up. However, when things look down, one may wish to forgo them so as to avoid unnecessary stress and discomfort.

## Author Contributions

SL, M-AV, and AS jointly developed the ideas for the project. SL and M-AV collected the data. SL analyzed the data. SL, M-AV, JZ, and AS prepared the manuscript.

## Conflict of Interest Statement

The authors declare that the research was conducted in the absence of any commercial or financial relationships that could be construed as a potential conflict of interest.
