# Supplementary material for: Better Not to Know? Emotion Regulation Fails to Benefit from Affective Cueing
Source: Front Hum Neurosci. 2016 Nov 25;10:599. doi: 10.3389/fnhum.2016.00599 (PMC5122596; doi:10.3389/fnhum.2016.00599)
Supplement: Supplementary file 1 [file Data_Sheet_1.DOC]

**Supplementary Material**

**PCA Component Selection**

PCA component (PC) selection was achieved using the following criteria.

- - 1. The selected temporal PC should explain more 1% of the variance. Seven temporal PCs for the cue epoch and five temporal PCs for the target epoch fulfilled this criterion. They are illustrated in supplementary Figures 1 and 2.


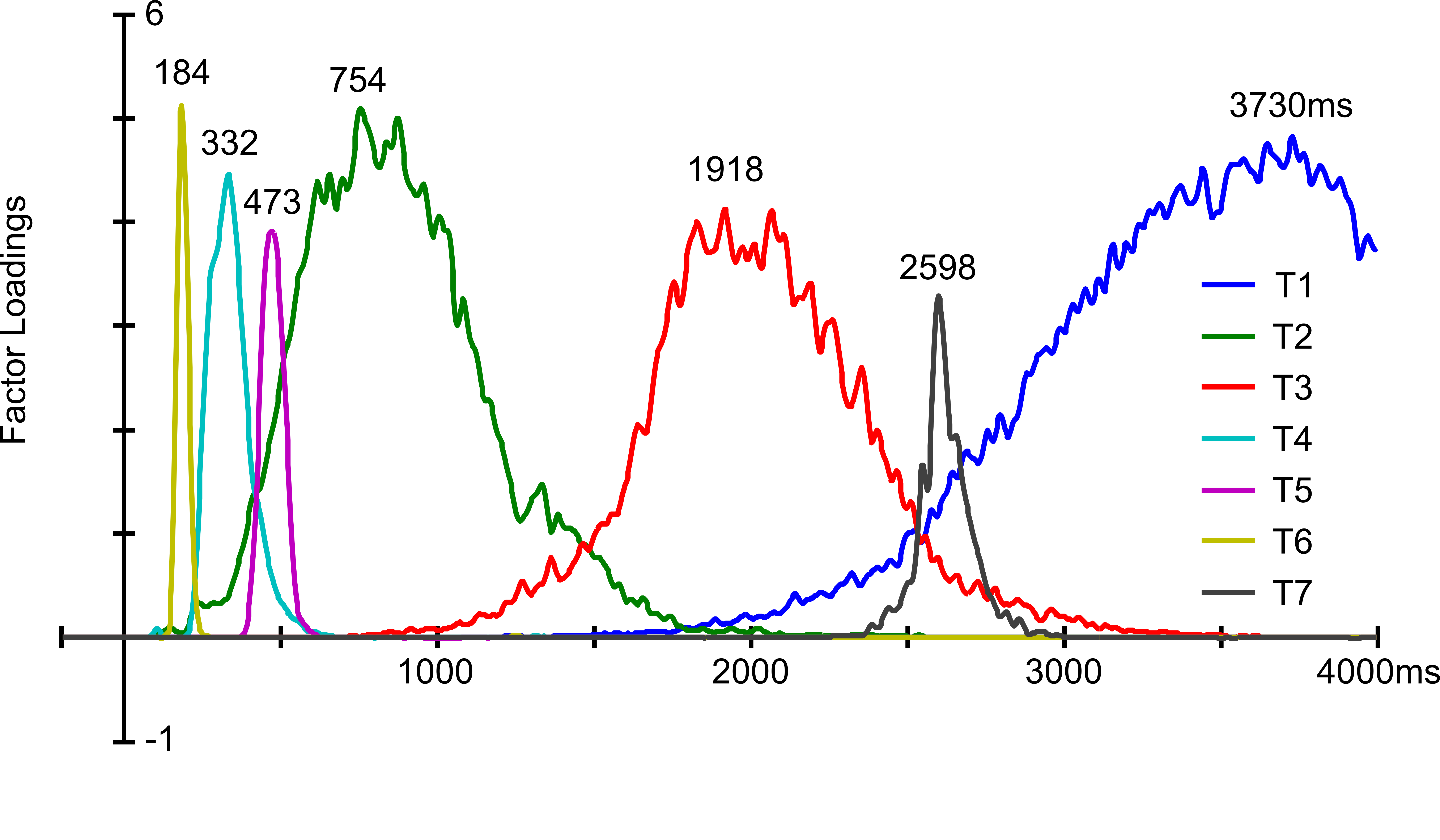
Supplementary Figure 1. Temporal distribution of components (T1 to T7) in cue epochs. Seven components were found explained more than 1% of variance, out of which three components (T2, T4, and T5) peaked in the 300-900ms time window. Peak latency of each component was also indicated in the figure.


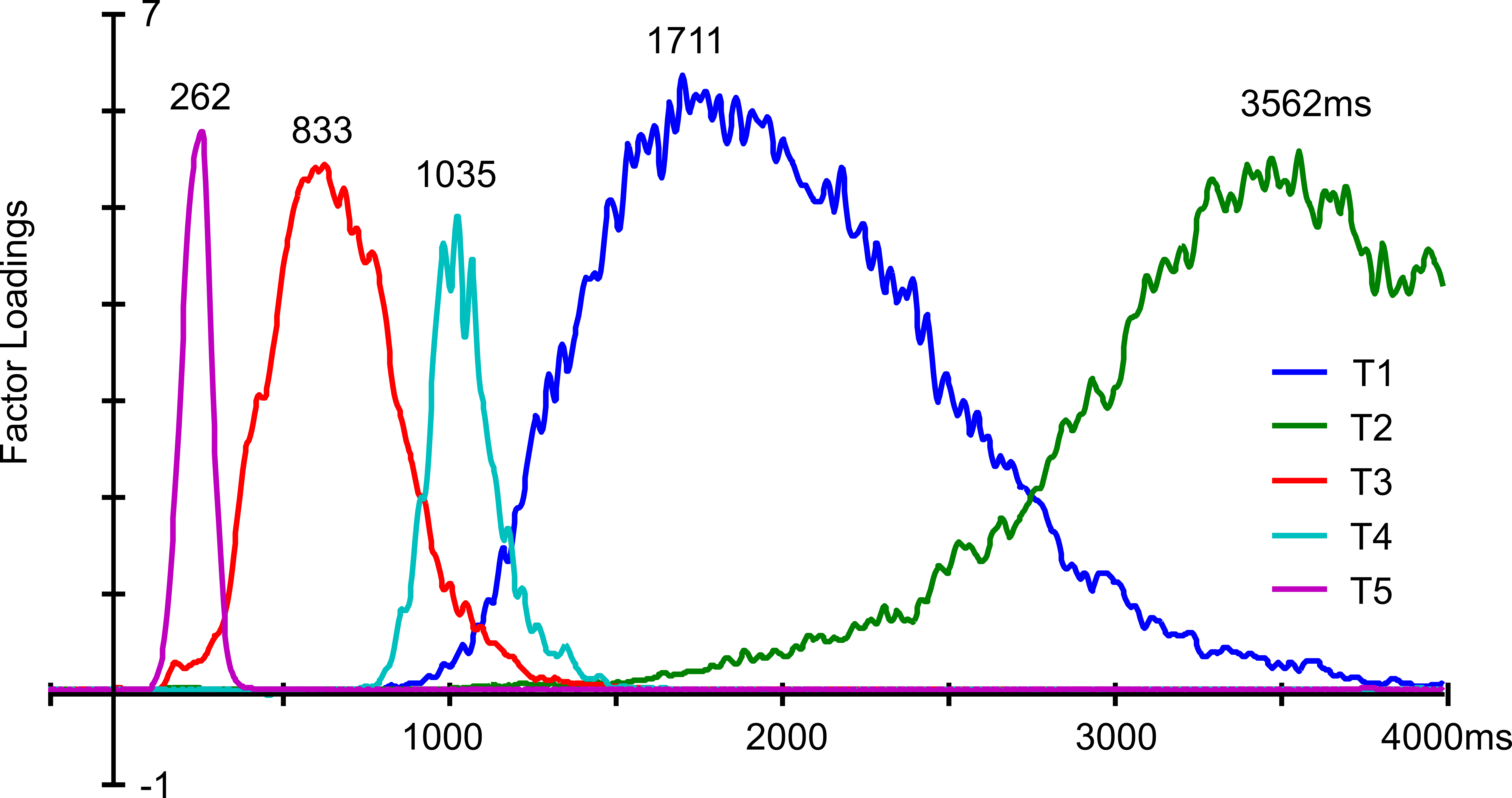
Supplementary Figure 2. Temporal distribution of components (T1 to T5) in target epochs. Five components were found explained more than 1% of variance, of which only T3 peaked in the 300-900ms time window. Peak latency of each component was also indicated in the figure.

(2) The selected temporal PCs should show a similar temporal course as the LPP and peak between 300 and 900 ms after cue/target onset. For the cue epoch, three temporal PCs fulfilled this criterion (i.e., T2, T4, and T5). For the target epoch, one temporal PC met this criterion (T3).

(3) We always selected the temopral-spatial PC that explained the largest variance because it should capture the dominant spatial distribution for a given time course. We obtained T2S1cue, T4S1cue and T5S1cue for the cue epoch and T3S1target for the target epoch.

(4) The selected temopral-spatial PCs should overlap in topography with the centro-pariatal topography of the LPP. T2S1cue, T5S1cue and T3S1target met this criterion. T4S1cue had an occipital scalp distribution and therefore was dropped from further analysis.

Statistical analyses were conducted on the components that fulfilled the four criteria above. Separate ANOVAs were performed on their factor scores with Appraisal (spontaneous, controlled), Affective Cueing (yes, no), and Target Valence (positive, negative) as independent variables. The Bonferoni method was applied to control for multiple comparisons. For the cue epoch, two PCs were identified. Thus, the significance level was adjusted to p < 0.025 (i.e., 0.05 divided by 2). For the target epoch, one PC was identified and thus the significance level remained at p < 0.05.

**Traditional ERP Analyses**

ERP analyses on the LPP were conducted for cue and target epochs separately. We subjected the average ERP amplitudes between 300 and 900ms following stimulus onset and across centro-parietal electrodes (C3/4, CP3/4, P3/4) to an ANOVA with Appraisal, Affective Cueing, and Target Valence as repeated measures factors.

For the cue epoch, the main effect of Affect Cueing was significant (F(1, 19) = 49.3, p < 0.001) as was the Affect Cueing by Target Valence interaction (F(1, 19) = 7.41, p = 0.01). Informative relative to the uninformative cues elicited a more positive LPP and this effect was larger in the negative (F(1, 19) = 53.1, p < 0.001) relative to the positive condition (F(1, 19) = 12.26, p < 0.01).

For the target epoch, the main effect of Affective Cueing was significant (F(1, 19) = 4.6, p = 0.045) as was the interaction of Appraisal, Affective Cueing, and Target Valence (F(1, 19) = 6.02, p = 0.024). Follow-up analyses were non-significant in the uninformative cue condition (p>.1). In the informative cue condition, Target Valence significantly interacted with Appraisal (F(1, 19) = 5.28, p = 0.03) indicating that controlled appraisal reduced LPP amplitudes relative to spontaneous appraisal for negative (F(1, 19) = 4.25, p = 0.05) but not positive targets (p < 0.1).
